# Comparing the income-related inequity of tested prevalence and self-reported prevalence of hypertension in China

**DOI:** 10.1186/s12939-018-0796-y

**Published:** 2018-06-15

**Authors:** Min Su, Yafei Si, Zhongliang Zhou, Chi Shen, Wanyue Dong, Xiaojing Fan, Xiao Wang, Xiaolin Wei

**Affiliations:** 1 School of Public Policy and Administration, Xi’an Jiaotong University, Xi’an, China; 2 School of Public Health, Xi’an Jiaotong University, Xi’an, China; 3 International Business School Suzhou, Xi’an Jiaotong-Liverpool University, Suzhou, China; 4 Dalla Lana School of Public Health, University of Toronto, Toronto, Canada

**Keywords:** China, Tested prevalence, Self-reported prevalence, Hypertension, Income-related health inequality, Decomposition, Horizontal inequity

## Abstract

**Background:**

Hypertension has become a global health challenge given its high prevalence and but low awareness and detection. Whether the actual prevalence of hypertension has been estimated is important, especially for the poor. This study aimed to measure tested prevalence and self-reported prevalence of hypertension and compare the inequity between them in China**.**

**Methods:**

Data were derived from China Health and Nutrition Survey (CHNS) conducted in 2011. By using the multistage, stratified, random sampling method, 12,168 respondents aged 18 or older were identified for analysis. Both tested prevalence (systolic blood pressure ≥ 140 mmHg or/and diastolic blood pressure ≥ 90 mmHg or /and current use any of antihypertensive medication) and self-reported prevalence (ever diagnosed with hypertension by a doctor) were used to measure the prevalence of hypertension. The concentration index was employed to measure the extent of inequality in tested prevalence and self-reported prevalence. A decomposition method, based on a Probit model, was used to analyze income-related horizontal inequity of tested prevalence and self-reported prevalence**.**

**Results:**

The tested prevalence and self-reported prevalence of total respondents were 28.8% [95% CI (28.0%, 29.6%)] and 15.7% [95% CI (15.0%, 16.3%)], and 26.4% [95% CI (25.1%, 27.6%)] and 19.0% [95% CI (17.9%, 20.1%)] in urban areas, and 30.3% [95% CI (29.3%, 31.4%)] and 13.5% [95% CI (12.7%, 14.3%)] in rural areas. The horizontal inequity indexes of mean tested prevalence and self-reported prevalence were − 0.0494 and 0.1203 of total respondents, − 0.0736 and 0.0748 in urban area, and − 0.0177 and 0.0466 in rural area respectively, indicating pro-poor inequity in tested prevalence and pro-rich inequity in self-reported prevalence of hypertension. Economic status, education attainment and age were key factors of the pro-poor inequity in tested prevalence. Economic status, area and age were key factors to explain the poor-rich inequity in self-reported prevalence.

**Conclusions:**

This study revealed self-reported prevalence of hypertension was much lower than tested prevalence in China, while a larger gap between self-reported and tested prevalence was found in rural areas. Our study suggested social strategies aiming at narrowing economic gap and regional disparities, reducing educational inequity, and facilitating health conditions of the elderly should be implemented. Finally, awareness raising campaigns to test hypertension in rural area need be strengthened by health education programs and improving the access to public health service, especially for those who do not engage with regular health checkups.

## Background

Hypertension has long been regarded as a crucial global health challenge given its high prevalence and leading risks for cardiovascular disease but low awareness and detection [1–3]. The past four decades have experienced a globally increasing number of population suffering from hypertension, which increased from 594 million in 1975 to 1.13 billion in 2015 world-wide [4, 5]. Residents living with hypertension may experience heavier burden of disease. As the GBD 2015 reported [5], hypertension has become the second risk factor accounting for 211.8 million disability-adjusted life-years (DALYs). Furthermore, residents with hypertension may be at a higher risk of incurring catastrophic health care expenditure especially for the poor [2]. Regarding the serious consequences, the awareness, detection, diagnosis and control of hypertension are significantly important for reducing the risks of hypertension. However, hypertension can be asymptomatic, and many patients living with hypertension may have not seen a doctor [6, 7]. Thus, it has become a crucial global health challenge for the awareness, detection and diagnosis of hypertension. By the end of 2011, there were approximately 28.6% of adults had hypertension in China and only 37.6% of them were diagnosed by a doctor [8]. Therefore, strategies aiming at improving the awareness, detection and diagnosis of hypertension and relieving the disease burden of residents with hypertension are essential.

Chinese government has carried out a wide range of health reforms to protect residents against hypertension since 2009, among which the basic free national public health program towards the achievement of universal health coverage is one of the most important policy interventions. The basic free public health services have been provided to the residents to establish a dynamic health record, screening those with hypertension or other chronic diseases for further monitoring, treating and controlling. In addition, annual free health check is available for each elderly people aged 65 or older, which also includes the screening of hypertension [9]. By the end of 2011, the proportion of people established health record in primary healthcare facilities accounted for over 50%, and more than 39 million aged people obtained health check free of charge [9–11].

Another intervention, the basic health insurance schemes, is also effective to release more demands of preventive care by the financial support for health care [12]. Thus, the residents with hypertension or other chronic diseases covered by more generous schemes are more likely to be diagnosed. There are three basic health insurance schemes in China: Urban Employee Basic Medical Insurance (UEBMI) for urban residents who work in formal sectors, Urban Resident Basic Medical Insurance (URBMI) for the rest urban residents without formal jobs or unemployment, and the New Rural Cooperative Medical Scheme (NRCMS) in rural areas [10]. The UEBMI, NRCMS and URBMI have been piloted in 1998, 2003 and 2007 respectively. In addition, there were approximately 252.3 million people enrolled in the UEBMI and 220.7 million people enrolled in the URBMI by the end of 2011, accounting for 36.5 and 31.9% of the total urban residents respectively. There were 0.8 billion people (accounting for 97.5% of the total rural residents) enrolled in the NRCMS by the end of 2011 [11]. However, the administration, pooling levels, and benefit packages are quite different across different schemes. For example, NRCMS involves a Household Account for critical outpatient care, such as stage III hypertension, whilst UEBMI sets up an Individual Account for more generous coverage for outpatient care [9].

Numerous studies have revealed that inequalities in socioeconomic status are associated with inequalities in health services and health outcomes. For example, higher economic and education level, good employment status, developed region, and having health insurance were all regarded as important factors of higher health service utilization and better health outcomes [12–15]. Furthermore, an increasing literature has shown lower economic and education level, absence of health insurance, and having chronic diseases (e.g. hypertension and diabetes) were associated with higher risk of inequality in catastrophic healthcare expenditure [3, 16]. Although China has made great progress in the management of hypertension and other chronic diseases, a key question is whether the actual prevalence of hypertension has been estimated, especially in the groups of lower socioeconomic status. Using different methods to measure the prevalence of hypertension and compare the income-related inequality between them can provide insights into the detection, awareness and diagnosis of hypertension. Prior research indicated that the self-reported measurements may result in an underestimation of the prevalence of hypertension primarily because many people are unaware of their conditions. However, most current studies only used tested measures or self-reported measures to reveal the prevalence of hypertension [16–20]. Therefore, measuring the inequality of hypertension prevalence is one of the most prevalent concerns for the entire health system. Given the strong disparity between tested prevalence and self-reported prevalence, the inequality of hypertension based on self-reported data may be less accurate than medical measurements. However, little evidence was available to measure and compare the income-related inequality between tested prevalence and self-reported prevalence of hypertension based on a large-scale survey of national households in China. Hence this study aimed to fill the gap by measuring tested prevalence and self-reported prevalence of hypertension and comparing the income-related inequality between them, and further decomposing the income-related inequality into the contributing factors, based on a nationally representative sample aged 18 years or older in 2011. This study will provide evidence-based strategies on reducing the income-related inequality of both tested and self-reported hypertension prevalence in China and other developing countries.

## Methods

### Ethics

The ethics approval was obtained by the review board from the University of North Carolina at Chapel Hill, National Institute for Nutrition and Food Safety, China Center for Disease Control and Prevention and China-Japan Friendship Hospital. Informed consent was obtained, and data were anonymized for the analysis.

### Data

Data were derived from the China Health and Nutrition Survey (CHNS) conducted in 2011. The CHNS, an international project, aimed to collect a nationally representative sample of Chinese residents to support the health and nutrition related research. The CHNS has been conducted every 2–4 years since 1989 [21, 22]. By using multistage, stratified, random sampling method, 9 primary sampling units of provinces/autonomous cities, 288 secondary units of communities and 5884 households were interviewed. Since this study focused on the respondents aged 18 years and older, 12,168 respondents were identified in the final sample for further analysis after data cleaning. Among those, urban respondents were 4791 (39.4%) and rural respondents were 7377 (60.6%). More details of the CHNS were available here (http://www.cpc.unc.edu/projects/chinak).

### Dependent variables

A subject was defined as having hypertension with two methods: tested prevalence and self-reported prevalence. For tested prevalence, mercury sphygmomanometers were used to measure each respondent’s systolic blood pressure (SBP) and diastolic blood pressure (DBP) three times after setting for 10 min. Then the mean SBP and DBP values were calculated. A subject was defined as having hypertension if he/she had an average SBP ≥ 140 mmHg or/and DBP ≥ 90 mmHg or current use of any anti-hypertensive medication, following the WHO guidelines [6, 22, 23]. For self-reported prevalence, the interviewees were asked: “Have you ever been diagnosed with hypertension by a doctor?” A subject was defined as having hypertension if he/she answered “Yes”.

### Independent variables

With reference to previous research [23–26], seven categories of factors, which may be related to the prevalence of hypertension were used in this study. Firstly, demographic characteristics and health statuses, including seven variables: gender, age, height, weight, educational achievement, marital status and self-reported diabetes status. BMI was calculated as weight divided by the square of the height (kg/m^2^). BMI were categorized as underweight (BMI < 18.5 kg/m^2^), normal weight (18.5 ≤ BMI < 25.0 kg/m^2^), overweight (25.0 ≤ BMI < 30.0 kg/m^2^) and obese (BMI ≥ 30.0 kg/m^2^) according to WHO classifications [3, 27]. The interviewees were asked: “Have you ever been diagnosed with diabetes by a doctor?” A subject was defined as having diabetes if he/she answered “Yes”. Secondly, utilization of preventative health service, including a dummy variable (e.g. whether receive preventative health service during the last 4 weeks). Thirdly, basic health insurance, including a dummy variable: having basic health insurance or not. Fourthly, economic status was measured by annual individual income, and grouped into five quintiles. Furthermore, geographic characteristic (area), consisting of 9 provinces including Liaoning, Heilongjiang, Jiangsu, Shandong, Henan, Hubei, Hunan, Guangxi and Guizhou, which were grouped into three areas (e.g. East, Middle and West of China). Finally, risk factors for hypertension, including two variables: smoking and drinking. Specially, respondents who ever consumed any form of tobacco were defined as smokers; respondents who consumed beer or any other alcoholic beverage last year were defined as drinkers. With reference to previous research, only variables passed the multicollinearity test were included, with none of the variables having a VIF greater than 4 [28].

### Health inequity

#### Concentration index

Concentration index was applied to quantify the income-related inequality in both tested prevalence and self-reported prevalence of hypertension [29, 30]. Concentration index ranges from − 1 to 1, and 0 represents no income-related inequality [31, 32]. The positive score of concentration index indicates pro-rich inequality in prevalence, and the negative score of concentration index indicates pro-poor inequality. The Eq.1 was used to calculate the concentration index:

1 $$ \mathrm{C}=\frac{2}{\mu}\mathit{\operatorname{cov}}\left(y,r\right) $$

Where C represents concentration index, *y* represents the prevalence of hypertension, μ represents the mean of the prevalence of hypertension, and γ represents the fractional rank of income distribution [33].

#### Decomposition methods of concentration index

The income-related inequality of tested prevalence and self-reported prevalence of hypertension could be explained by decomposing concentration index into the contributing factors based on a Probit model, using Eq.2 and Eq.3 [34]. As the Probit model is a nonlinear model, the linear approximation to the nonlinear model was conducted by estimating the partial effects evaluated at the covariate means.

2 $$ {y}_i=\alpha +{\sum}_j{\beta}_j^m{x}_{ji}+{\sum}_k{r}_k^n{z}_{ki}+{\varepsilon}_i\kern0.75em $$

where *y*_*i*_ represents the prevalence of hypertension; χ represents the need factors of hypertension prevalence. Need factors are the unavoidable determinants of hypertension prevalence. As Wagstaff and Van Doorslaer suggested, gender and age are commonly used to reflect need factors of health [15, 16, 35]; z represents the non-need factors of hypertension prevalence. Non-need factors are the avoidable determinants of hypertension prevalence, including annual individual income, educational states, marital states, BMI, diabetes, smoke, drink, area, basic health insurance and utilization of preventative health service, following the literature [15, 16, 35]; ${\beta}_j^m$ and ${r}_k^n$ are marginal effects of each variable; *ε*_*i*_ stands for the error. The concentration index C could also be written as:

3 $$ \mathrm{C}={\sum}_j\left(\raisebox{1ex}{${\beta}_j^m\overline{x_j}$}\!\left/ \!\raisebox{-1ex}{$\mu $}\right.\right){C}_j+\raisebox{1ex}{${\mathrm{G}C}_{\varepsilon }$}\!\left/ \!\raisebox{-1ex}{$\mu $}\right. $$

Where μ is the mean of the prevalence of hypertension, *C*_*j*_ are the concentration index for *x*_*j*_ and $\overline{x_j}$ are the means of *x*_*j*_. The contributions of independent variables are indicated by the first item on the right side of Eq.3 to the inequality of prevalence indicators, the last term is the generalized concentration index of ε. From Eq.3, we can see that the overall inequality can be divided into two parts: explained part calculated by the first term and unexplained part calculated by the last term [33].

#### Horizontal inequity index

The horizontal inequity index of health, which indicates the inequality of health for the people who have the same unavoidable health conditions, was measured by deducting the contributions of need factors from the concentration index of health [35, 36]. In this study, the horizontal inequity index of hypertension prevalence was obtained by removing the contributions of need factors (gender and age) from the overall concentration index of hypertension prevalence. A positive (negative) horizontal inequity index indicated the pro-rich (pro-poor) inequity. All analyses were performed in Stata 13.0.

## Results

### Descriptive analysis

In 2011, among the whole respondents (12,168), the annual individual income was RMB 17,012 (urban: RMB 21,567; rural: RMB 14,060). More than half of the total respondents tended to be females and the percentages of female and male respondents in both urban and rural areas were roughly equal. Majority of respondents had normal weight, had an education level at secondary school, have been diagnosed with diabetes by a physician, never smoke and drink, had basic health insurance, and received preventative health service in the last 4 weeks. More details can be found in Table 1.

**Table 1:** Description of independent variables in 2011

Variables	Description of variables	Total N (%)	Urban N (%)	Rural N (%)
Gender				
Male^a^		5757(47.31)	2272(47.42)	3485(47.24)
Female	Male = 0, female = 1	6411(52.69)	2519(52.58)	3892(52.76)
Age group				
18–44^a^		4346(35.72)	1658(34.61)	2688(36.44)
45–59	45–59 = 1, else = 0	4038(33.19)	1566(32.69)	2472(33.51)
> 60	60 and above = 1, else = 0	3784(31.10)	1567(32.71)	2217(30.05)
BMI				
Underweight^a^	BMI < 18.5 kg/m^2^	621(5.18)	222(4.69)	399(5.49)
Normal weight	18.5 ≤ BMI < 25.0 kg/m^2^ = 1, else = 0	5864(48.88)	2272(48.04)	3592(49.42)
Overweight	25.0 ≤ BMI < 30.0 kg/m^2^ = 1, else = 0	3756(31.31)	1529(32.33)	2227(30.64)
Obese	BMI ≥ 30.0 kg/m^2^ = 1, else = 0	1756(14.64)	706(14.93)	1050(14.45)
Diabetes				
No^a^		11,673(95.93)	4496(96.84)	7177(97.27)
Yes	Have been diagnosed with diabetes by a doctor =1, else = 0	482(3.96)	285(5.95)	197(2.67)
Education				
Illiterate or Primary^a^		4338(35.70)	1063(22.23)	3275(44.44)
Secondary	Secondary =1, else = 0	6326(52.07)	2699(56.45)	3627(49.22)
College or above	College degree or above =1, else = 0	1486(12.23)	1019(21.31)	467(6.34)
Marital status				
Single^a^		641(5.36)	339(7.21)	302(4.16)
Marriage	Marriage =1, else = 0	10,108(84.53)	3876(82.42)	6232(85.90)
Others	Others =1, else = 0	1209(10.11)	488(10.38)	721(9.94)
Smoke				
No^a^	Never smoke	8463(69.55)	3386(70.67)	5077(68.82)
Yes	Smoke = 1, else = 0	3701(30.42)	1403(29.28)	2298(31.15)
Drink				
No^a^	Never drink	8048(66.14)	3011(62.85)	5037(68.28)
Yes	Drink beer or alcohol last year = 1, else = 0	4118(33.84)	1778(37.11)	2340(31.72)
Area				
East of China^a^	Living in the east of China	5188(42.64)	2359(49.24)	2829(38.35)
Middle of China	Living in the middle of China = 1, else = 0	5058(41.57)	1644(34.31)	3414(46.28)
West of China	Living in the west of China = 1, else = 0	1922(15.80)	788(16.45)	1134(15.37)
Basic health insurance				
No^a^	Absence basic health insurance	414(3.40)	205(4.28)	209(2.83)
Yes	Having basic health insurance = 1, else = 0	11,751(96.60)	4583(95.72)	7168(97.17)
Preventative health service				
No^a^		901(7.40)	448(9.35)	453(6.14)
Yes	Receiving preventative health service in the last four weeks = 1, else = 0	11,255(92.50)	4339(90.57)	6916(93.75)
Economic status				
Quintile1 (poorest^a^)	20% low income individuals	2429(19.96)	957(19.97)	1474(20.01)
Quintile2 (poorer)	20% lower income individuals = 1, else = 0	2431(19.98)	954(19.91)	1477(20.05)
Quintile3 (middle)	20% middle-income individuals = 1, else = 0	2424(19.92)	955(19.93)	1470(19.96)
Quintile4 (richer)	20% higher income individuals = 1, else = 0	2429(19.96)	954(19.91)	1473(20.00)
Quintile5 (richest)	20% high income individuals = 1, else = 0	2426(19.94)	953(19.89)	1472(19.98)
Observations		12,168	4791	7377

Note: ^a^Reference levels in the probit regression. Only variables passed the multicollinearity test were included, with none of the variables having a VIF greater than 4

### Mean SBP, mean DBP, tested prevalence and self-reported prevalence

The mean SBP of total respondents was 124.4 ± 17.7 mmHg (urban: 124.1 ± 17.0 mmHg; rural: 124.6 ± 17.2 mmHg) and the mean DBP was 79.2 ± 10.7 mmHg (urban: 78.8 ± 9.9 mmHg; rural: 79.5 ± 11.2 mmHg). The tested prevalence and self-reported prevalence of total respondents were 28.8% [95% CI (28.0%, 29.6%)] and 15.7% [95% CI (15.0%, 16.3%)], and 26.4% [95% CI (25.1%, 27.6%)] and 19.0% [95% CI (17.9%, 20.1%)] in urban areas, and 30.3% [95% CI (29.3%, 31.4%)] and 13.5% [95% CI (12.7%, 14.3%)] in rural areas (Table 2). It was notable significant differences between tested prevalence and self-reported prevalence of total respondents were observed (both urban and rural areas) at the level of α = 0.05. It was also worth noting the gap between tested prevalence and self-reported prevalence of total population, urban residents and rural respondents was 13.1%, 7.4% and 16.8% respectively.

**Table 2:** Tested prevalence and self-reported prevalence of hypertension in different economic quantiles, 2011 (%)

	Total		Urban		Rural	
	Tested prevalence	Self-reported prevalence	Tested prevalence	Self-reported prevalence	Tested prevalence	Self-reported prevalence
Quintile 1 (poorest)	33.10 (31.23, 34.97)	13.42 (12.06, 14.78)	31.77 (28.81, 34.72)	15.88 (13.56, 18.20)	34.12 (31.70, 36.55)	13.30 (11.56, 15.03)
Quintile 2 (poorer)	29.41 (27.60, 31.22)	13.04 (11.70, 14.38)	26.31 (23.51, 29.11)	15.20 (12.92, 17.48)	30.87 (28.51, 33.23)	11.65 (10.00, 13.28)
Quintile 3 (middle)	28.18 (26.38, 29.97)	14.07 (12.68, 15.45)	26.39 (23.59, 29.19)	18.01 (15.57, 20.45)	30.14 (27.79, 32.48)	13.33 (11.59, 15.07)
Quintile 4 (richer)	27.79 (26.01, 29.57)	17.83 (16.30, 19.35)	26.62 (22.60, 28.13)	22.96 (20.28, 25.63)	28.45 (26.14, 30.75)	13.17 (11.44, 14.49)
Quintile 5 (richest)	25.23 (23.50, 26.96)	19.95 (18.36, 21.50)	21.39 (19.30, 24.56)	22.98 (20.30, 25.66)	27.85 (25.65, 30.15)	16.30 (14.16, 17.91)
Total	28.76 (27.96, 27.67)	15.68 (15.03, 16.33)	26.38 (25.13, 27.63)	19.01 (17.90, 20.13)	30.30 (29.26, 31.36)	13.51 (12.73, 14.30)

Note: Mean and 95% CI (in parentheses) were reported

Table 2 also presents tested prevalence and self-reported prevalence of hypertension in different economic quantiles. In terms of tested prevalence, the poorest had the highest probability of hypertension [33.1%, 95% CI (31.2%, 35.0%)], while the richest had the lowest probability of hypertension [25.2%, 95% CI (23.5%, 27.0%)], and the trend was consistent in both urban and rural areas. However, the self-reported prevalence indicated an increase from the poorest [13.4%, 95% CI (12.1%, 14.8%)] to the richest [19.9%, 95% CI (18.4%, 21.5%)], and the trend was also consistent in both urban and rural areas.

### Concentration index and decomposition

#### Concentration index

The income-related inequalities of tested prevalence for the whole population, urban and rural areas were − 0.0544 [95% CI (− 0.0705, − 0.0383)], − 0.0626 [95% CI (− 0.0899, − 0.0353)], and − 0.0367 [95% CI (− 0.0567, − 0.0167)] respectively, which meant that the poor had a higher prevalence of hypertension based on the tested measurement. However, the income-related inequalities of self-reported prevalence of hypertension for the whole population, urban and rural areas were 0.0888 [95% CI (0.0551, 0.0225)], 0.0436 [95% CI (0.0103, 0.0769)], and 0.0893 [95% CI (0.0656, 0.1131)] respectively, which meant that the rich had a higher self-reported prevalence of hypertension.

#### Decomposition of inequality in tested prevalence and self-reported prevalence

The decomposition of concentration index of the tested prevalence and self-reported prevalence of hypertension are presented in Tables 3 and 4 respectively. The estimated partial effects of related factors of inequality in tested prevalence and self-reported prevalence of hypertension were calculated based on Probit model. Contribution to the inequality and the proportion of contribution in the overall concentration index are also reported. A negative marginal effect of annual individual income, taking the tested prevalence of total respondents for example, indicated a higher income level was significantly associated with lower tested prevalence, whilst a positive marginal effect of age for instance, suggested the tested prevalence increased along the ageing population.

**Table 3:** Partial effect and contribution to concentration index(Tested prevalence)

Variables	Total			Urban			Rural		
	dy/dx	Con	%	dy/dx	Con	%	dy/dx	Con	%
Annual individual income			55.98			59.71			50.56
Quintile2 (poorer)	−0.0189	0.005	−9.69	−0.0327	0.010	−15.80	− 0.0112	0.002	− 6.37
Quintile3 (middle)	− 0.0210	0.000	0.01	− 0.0320	0.000	0.03	− 0.0104	− 0.001	3.75
Quintile4 (richer)	−0.0283^*^	− 0.008	14.47	− 0.0420^*^	− 0.013	20.39	− 0.0113	− 0.004	9.97
Quintile5 (richest)	− 0.0501^***^	− 0.028	51.19	− 0.0569^**^	− 0.034	55.09	− 0.0429	− 0.016	43.21
Female	0.0756^***^	0.003	−5.37	0.0837^***^	0.002	−3.68	0.0644^***^	0.003	−8.06
Age(years)			14.16			−12.89			60.26
45–59	0.1590^***^	0.007	−13.56	0.1155^***^	−0.001	2.21	0.1891^***^	0.016	−43.62
> 60	0.3002^***^	−0.015	27.72	0.2727^***^	0.010	−15.10	0.3293^***^	−0.038	103.88
BMI			−10.06			19.80			−34.83
Normal weight	0.1642^***^	−0.003	4.84	0.1583^**^	0.004	−6.29	0.1706^***^	−0.005	12.86
Overweight	0.3121^***^	0.019	−34.64	0.2938^***^	0.011	−17.15	0.3273^***^	0.021	−57.99
Obese	0.5764^***^	−0.011	19.74	0.5675^***^	−0.027	43.24	0.5833^***^	−0.004	10.30
Diabetes	0.0609^***^	0.001	−2.54	0.0620^*^	0.001	−2.12	0.0779^*^	0.001	−2.09
Education			46.41			38.62			37.53
Secondary	−0.0571^***^	−0.005	8.71	−0.0476^***^	0.002	−3.04	−0.0454^**^	− 0.005	14.65
College or above	−0.0992^***^	−0.020	37.70	−0.0850^***^	− 0.026	41.66	− 0.0837^**^	−0.008	22.92
Marital status			4.08			2.33			10.28
Marriage	−0.0083	0.000	0.48	0.0163	0.000	−0.68	−0.0333	− 0.002	4.89
Others	0.0392	−0.002	3.60	0.0447	−0.002	3.01	0.0331	−0.002	5.39
Smoke	0.0049	0.000	0.09	−0.0100	0.000	−0.77	0.0144	0.000	−0.70
Drink	0.0267^*^	0.002	−3.33	0.0233	0.000	−0.70	0.0317^*^	0.002	−6.54
Area			−0.73			6.29			−7.39
Middle of China	0.0096	−0.002	4.13	0.0321^*^	−0.007	11.73	−0.0049	0.001	−2.60
West of China	−0.0281^*^	0.003	−4.86	−0.0209	0.003	−5.44	−0.0281	0.002	−4.79
Basic insurance	0.0359	−0.002	3.15	0.0317	−0.002	2.71	0.0444	−0.002	4.93
Preventative health service	0.0288^*^	0.001	−2.48	0.0258^*^	0.002	−2.27	0.0393	0.001	−3.34

Note: *dy/dx* the partial effect in Probit regression model, *Con* the contribution to inequality; %, the share of contribution index. ^*^P < 0.05; ^**^P < 0.01; ^***^P < 0.001

**Table 4:** Partial effect and contribution to concentration index (Self-reported prevalence)

Variables	Total			Urban			Rural		
	dy/dx	Con	%	dy/dx	Con	%	dy/dx	Con	%
Annual individual income			56.27			48.23			91.73
Quintile2 (poorer)	0.0069	−0.004	−3.95	−0.0141	0.006	6.65	0.0211^*^	−0.010	−22.75
Quintile3 (middle)	0.0111	0.000	0.01	−0.0136	0.000	−0.01	0.0216^*^	0.006	14.68
Quintile4 (richer)	0.0243^*^	0.012	13.91	0.0166	0.007	7.87	0.0248^*^	0.018	41.21
Quintile5 (richest)	0.0406^***^	0.041	46.30	0.0356	0.030	33.72	0.0308^*^	0.026	58.59
Female	0.0020	0.000	0.15	0.0104	0.000	0.45	−0.0019	0.000	−0.45
Age(years)			−16.19			15.16			−7.48
45–59	0.1742^***^	0.015	16.60	0.1988^***^	−0.003	−3.72	0.1562^***^	0.0300	67.98
> 60	0.3180^***^	−0.046	−32.79	0.3487^***^	0.017	18.88	0.2851^***^	−0.033	−75.46
BMI			7.27			−4.61			26.59
Normal weight	0.0582^***^	−0.002	−1.91	0.1222^***^	0.004	4.75	0.0298^*^	−0.002	− 4.24
Overweight	0.1499^***^	0.017	18.58	0.2271^***^	0.012	12.97	0.1147^***^	0.017	38.31
Obese	0.2456^***^	−0.008	−9.40	0.0998^***^	−0.020	−22.33	0.2245^***^	−0.003	−7.48
Diabetes	0.1461^***^	0.006	6.81	0.1834^***^	0.005	6.13	0.1030^***^	0.0023	5.21
Education			−13.34			−21.97			−23.52
Secondary	−0.0173^*^	−0.003	−2.94	−0.0238	0.001	1.48	−0.0239^**^	−0.006	−14.58
College or above	−0.0245^*^	−0.009	−10.40	− 0.0489^**^	−0.021	−23.45	− 0.0173	−0.004	−8.94
Marital status			−12.84			−7.08			−45.50
Marriage	0.0774^***^	0.005	5.10	0.0750^*^	0.003	3.05	0.0834^**^	0.010	23.13
Others	0.1748^***^	−0.016	−17.94	0.1539^**^	−0.009	−10.13	0.2235^**^	−0.030	−68.63
Smoke	−0.0012	0.000	0.02	−0.0010	0.000	0.07	0.0007	0.000	0.07
Drink	−0.0022	0.000	−0.31	−0.0116	0.000	−0.34	0.0034	0.000	1.32
Area			29.93			37.99			43.42
Middle of China	−0.0395^***^	0.017	18.94	−0.0523^***^	0.017	18.71	−0.0284^***^	0.013	28.67
West of China	−0.0569^***^	0.010	10.99	−0.0759^***^	0.018	19.28	−0.0460^***^	0.006	14.75
Basic insurance	−0.0019	0.000	0.19	0.0052	0.000	−0.43	−0.0123	0.001	2.58
Preventative health service	0.0936^***^	0.008	8.99	0.0927^***^	0.007	7.97	0.0938^***^	0.007	15.05

Note: *dy/dx* the partial effect in Probit regression model, *Con* the contribution to inequality; %, the share of contribution index. ^*^P < 0.05; ^**^P < 0.01; ^***^P < 0.001

After decomposing the concentration index, the income-related inequality was decomposed into the contribution of different variables. And a positive (negative) contribution represented the variable raised (reduced) the inequality [34]. It indicated that majority of the inequality of tested hypertension prevalence of total respondents were attributable to economic status (56.0%), education attainment (46.1%) and age (14.2%) by defining the contribution as proportions of each variable. It also indicated that majority of the inequality of tested hypertension prevalence in urban areas were attributable to economic status (59.7%), education attainment (38.6%) and BMI (19.8%). And the income-related in the tested prevalence of hypertension in rural areas were mainly explained by age (60.3%), economic status (50.6%) and education attainment (37.5%). In terms of self-reported prevalence, the majority of the inequality of self-reported hypertension prevalence of total respondents were attributable to economic status (56.3%), area (29.9%) and age (− 16.2%). In the urban areas, most of the inequality of self-reported hypertension prevalence were attributable to economic status (48.2%), area (38.0%) and education attainment (− 22.0%). And the income-related in the self-reported prevalence of hypertension in the rural areas were mainly explained by economic status (91.7%), marital status (− 45.5%), and area (43.4%).

#### Horizontal inequity index

The horizontal inequity index of tested prevalence and self-reported prevalence of hypertension are presented in Table 5. The horizontal inequity index of tested prevalence and self-reported prevalence of hypertension were − 0.0494 and 0.1203 of total respondents, − 0.0736 and 0.0748 in urban area, and − 0.0177 and 0.0466 in rural area respectively.

**Table 5:** Contributions and horizontal inequity of tested prevalence and self-reported prevalence of hypertension

	Total				Urban				Rural			
	Tested		Self-reported		Tested		Self-reported		Tested		Self-reported	
	Con	%	Con	%	Con	%	Con	%	Con	%	Con	%
Need factors												
Age-gender	−0.0050	8.79	−0.0310	−16.04	0.0110	−16.57	0.0140	15.61	−0.0190	52.20	−0.0030	−7.93
Non-need factors												
Economic level	−0.0310	55.98	0.0490	56.27	−0.0370	59.71	0.0430	48.23	−0.0190	50.56	0.0400	91.97
Other factors	−0.0190	33.92	0.0025	19.90	−0.0410	63.89	0.0170	11.60	0.000	−2.15	0.0113	25.22
Residual	0.0004	1.31	0.0688	39.87	0.0044	−7.03	0.0148	24.56	0.0013	−4.91	−0.0047	−9.26
CI	−0.0544	100	0.0893	100	−0.0626	100	0.0888	100	−0.0367	100	0.0436	100
HI	−0.0494		0.1203		−0.0736		0.0748		−0.0177		0.0466	

Note: *CI* concentration index, *HI* horizontal inequity index

## Discussions

### Self-reported prevalence and tested prevalence of hypertension

We investigated the prevalence of hypertension as well as its income-related inequality in China by using the data from the China Health and Nutrition Survey (CHNS) conducted in 2011. The results of this study revealed that self-reported prevalence of hypertension was much lower than tested prevalence in China. And similar trends were observed in both rural and urban areas. However, a larger gap between tested prevalence and self-reported prevalence was found in rural areas (16.8%). It’s worth noting that the gap between these two measurements in rural areas may be larger than 16.8% because we only focused on the current use of any anti-hypertensive medication. However, previous studies have revealed that 20% of patients who ever taken drugs stopped drugs at least a month [36]. Compared with urban residents, there are many challenges in having proper anti-hypertensive drugs in rural areas because of the limited qualification of physicians, heavy reliance on traditional therapies and the cost of such drugs [36–38]. It has been demonstrated that nearly 50% of hypertension patients were not receiving any anti-hypertensive medication in a national survey and only 24.6% in Yunnan province and 46.34% in Zhejiang province [37, 39–41].

Our results were comparable to those found in a prior study that used the same data in 2011 to measure the prevalence and awareness in Chinese adults. In that study, approximately 28.6% of Chinese adults had hypertension in 2011 [26]. Our results indicated a similar tested prevalence (28.8% for the total respondents, 26.4% in urban areas and 30.3% in rural areas) and an extremely low self-reported prevalence of hypertension in China (15.7% for the total respondents, 19.0% in urban areas and 13.5% in rural areas), which showed a low-level diagnosis and awareness of hypertension. It was also consistent with other international studies that there existed large gap between self-reported prevalence and tested prevalence in other low-income and middle-income countries (e.g. nearly 31% in Ghana, 10% in India, 40% in South Africa, and 16% in Mexico) [42].

### Poor-rich inequity in self-reported prevalence and poor-poor inequity in tested prevalence

The negative concentration index in tested prevalence of hypertension revealed that the poor tended to have higher tested prevalence than the rich, while the positive concentration index in self-reported prevalence of hypertension revealed that the rich people tended to have higher self-reported prevalence of hypertension than the poor, which was consistent with other low-income and middle-income countries. For example, the concentration index of self-reported and tested prevalence of hypertension was 0.02 and -0.07 in Russia, 0.14 and -0.02 in South Africa, and 0.19 and 0.03 in India, respectively. [42].

By removing the contributions of need factors (gender and age) from the overall concentration index, we compared the horizontal inequity index of tested prevalence and self-reported prevalence. The negative horizontal inequity index of tested prevalence revealed that controlling the unavoidable characteristics of respondents living with hypertension, there still existed the pro-poor inequity of prevalence. However, the positive horizontal inequity index of self-reported prevalence revealed that there existed the pro-rich inequity of prevalence.

The significant difference between tested prevalence and self-reported prevalence of hypertension, and inequity between them should be noted. If self-reports were used to measure the prevalence of hypertension, we may get unreal or extremely opposite conclusion that the rich tended to have high prevalence of hypertension. One potential factor could be responsible for this phenomenon. In 2011, an estimated less than 50% of adults aged 65 or older obtained health check although annual free health check is available for each elderly people aged 65 or older. And another public intervention was established that outpatients aged 35 or older are required to measure blood pressure at first when they seek outpatient care. However, the low self-reported prevalence indicated that there was a big problem in the access and utilization of preventative health service or other public health programs, which resulted in a big detection, awareness and management of major non-communicable diseases [9, 23]. In other words, the self-reported prevalence was more likely to reflect the accessibility of healthcare and public health programs other than the actual prevalence. Therefore, tested measurements were more reliable to estimate the prevalence of hypertension. Given hypertension is the determining risk factor for cardiovascular diseases and cerebrovascular diseases, our results highlighted the need to take positive measures to improve basic public health service system to screen those with hypertension or other chronic diseases for further monitoring, treating and controlling, including perfecting free health check program, establishing a dynamic health record and improving the quality of family doctors to ensure the longitudinal use of primary healthcare and preventative health service over time, and optimizing health education program and interventions. Based on the tested prevalence, this study indicated that the poor tended to have high prevalence of hypertension, thus further policy interventions aimed at improving the detection, awareness and management of hypertension and other chronic diseases should be taken to address the remaining pro-poor inequity.

### Decomposition of inequality in tested prevalence and self-reported prevalence

Results from the decomposition suggested that economic status, education attainment and age were the key factors of the pro-poor inequality in tested prevalence. Take the total respondents for example, the total contributions of economic status and education attainment were 102.4% with negative association with tested prevalence and negative positive contribution, highlighting that the poorer and/or less educated individuals were more likely to have hypertension. One potential reason to explain this was that the richer or more educated respondents may have more knowledge and better health awareness about addressing risk factors of hypertension, such as unhealthy diet, harmful use of alcohol and lack of physical activity [25]. Age was the third largest factor with positive association with tested prevalence, highlighting that the older people have higher prevalence of hypertension. In terms of the self-reported prevalence, the total contributions of economic status and area were 86.2% with positive association with self-reported prevalence and positive contribution, highlighting that the richer and/or individuals who live in the eastern of China were more likely to have higher self-reported of hypertension. One potential reason to explain this was that the richer or individuals from eastern China (eastern China is the most developed part of China with higher per capita income and more advanced health system) could benefit more from health system and public health programs, owning to the higher economic level, better accessibility of healthcare and chronic disease management system. Age was the third largest factor with positive association with self-reported prevalence, highlighting that the older people have higher self-reported prevalence of hypertension. Not surprisingly, elderly people were more likely to have unfavorable health outcomes and thus more utilization of preventative health service or other public health programs. Finally, the results from decomposing of income-related inequality suggested it was necessary to consider the contributions of key determinants when formulating health policy interventions, allocating health resources and relieving health inequity. It is crucial to facilitate the health conditions of the aged population and narrow economic, educational and geographical gaps between the rich and the poor in both rural and urban residents. Our research highlighted several recommendations that might be helpful to narrow the healthcare service and health outcome gaps. First, more attention should be paid to combine health policy with all other major policies, especially with the poverty reduction policies (Health Assistance Program for Poverty Alleviation) [43, 44]. Specifically, the health burdens of the poverty-stricken population should be alleviated by improving health insurance level. Furthermore, a triage system to the poor population with chronic diseases should be strengthened by establishing a dynamically electric health file and a health card. Adopting the mechanism of providing diagnosis and treatment before payment for the poor in cities or provinces where conditions permit would improve accessibility to critical healthcare [44, 45]. Second, more attention should be paid to the uneven geographic distribution in healthcare resources between urban and rural areas. Density of healthcare resources is significantly important for improving population health [46, 47]. Equitable geographic distribution of healthcare resources is proven to be associated with equities in health outcomes [48]. The distribution of health resources was highly spatially clustered in China [48–51]. For example, the number of licensed doctors per 1000 registered population was 3.5 in urban areas in 2014, which was 2.3 times of rural areas [51]. The number of beds per 1000 people was 6.9 in urban areas, and 3.1 in rural areas [50]. To reduce the uneven geographic distribution in healthcare resources between urban and rural areas, the health funding levels, sources and mechanisms should be adjusted and optimized, with an aim of reducing the urban and rural gap [48].

### Strengths and limitations

To sum up, our study has two key strengths. The first one is the large sample that is nationally representative, implying the conclusion from this study could be generalizable for the entire China. Apart from this, this is the first study to compare the inequity between tested prevalence and self-reported prevalence of hypertension and decompose into its contributing factors to explain health inequality based on a large-scale national household survey in China. This study will provide recommendations with an evidence-based approach for reducing the income-related inequality of hypertension prevalence and diagnosis in China and other developing countries. Two main limitations should also be noted in this study. Firstly, owing to the cross-sectional data, only correlation other than causality was investigated. Secondly, because of the data availability, we could not include all the unobservable variables. For example, dietary intake, work-related physical activity and comorbidity such as cardiovascular disease was not discussed in our study. The omission of these factors could lead to biased estimation of the inequality of hypertension prevalence.

## Conclusions

This study revealed self-reported prevalence of hypertension was much lower than tested prevalence in China, while a larger gap between self-reported and tested prevalence was found in rural areas. Furthermore, there existed pro-poor inequity in tested prevalence and pro-rich inequity in self-reported prevalence of hypertension. Our studies suggested social strategies aiming at narrowing economic gap and regional disparities, reducing educational inequity, and facilitating health conditions of the elderly should be implemented. Finally, awareness raising campaigns to test hypertension in rural area need be strengthened by health education programs and improving the access to public health service, especially for those who do not engage with regular health checkups.

## Abbreviations

CI: Concentration Index
DBP: Diastolic blood pressure
HI: Horizontal inequity index
SBP: systolic blood pressure
